# An Overview of Peripheral Blood Mononuclear Cells as a Model for Immunological Research of *Toxoplasma gondii* and Other Apicomplexan Parasites

**DOI:** 10.3389/fcimb.2019.00024

**Published:** 2019-02-08

**Authors:** John Alejandro Acosta Davila, Alejandro Hernandez De Los Rios

**Affiliations:** GEPAMOL, Centro de Investigaciones Biomédicas, Universidad del Quindío, Armenia, Colombia

**Keywords:** PBMCs (peripheral blood mononuclear cells), immunologic research, toxoplasma gondii, model of study, apicomplexa

## Abstract

In biology, models are experimental systems meant to recreate aspects of diseases or human tissue with the goal of generating inferences and approximations that can contribute to the resolution of specific biological problems. Although there are many models for studying intracellular parasites, their data have produced critical contradictions, especially in immunological assays. Peripheral blood mononuclear cells (PBMCs) represent an attractive tissue source in pharmacogenomics and in molecular and immunologic studies, as these cells are easily collected from patients and can serve as sentinel tissue for monitoring physiological perturbations due to disease. However, these cells are a very sensitive model due to variables such as temperature, type of stimulus and time of collection as part of posterior processes. PBMCs have been used to study *Toxoplasma gondii* and other apicomplexan parasites. For instance, this model is frequently used in new therapies or vaccines that use peptides or recombinant proteins derived from the parasite. The immune response to *T. gondii* is highly variable, so it may be necessary to refine this cellular model. This mini review highlights the major approaches in which PBMCs are used as a model of study for *T. gondii* and other apicomplexan parasites. The variables related to this model have significant implications for data interpretation and conclusions related to host-parasite interaction.

## Introduction

The phylum Apicomplexa consists of approximately 6,000 species of intracellular protozoan parasites, including various important human and animal pathogens such as *Plasmodium*, the causative agent of malaria; *Cryptosporidium*, the causative agent of cryptosporidiosis; *Theileria, Babesia* and *Eimeria*, which are important pathogens in cattle and fowl; and *T. gondii*, which is responsible for toxoplasmosis in birds, marsupials and mammals including humans (Tenter et al., 2000; Dubey, 2010). *T. gondii* has emerged as a model system for the study of intracellular parasitism; it is one of the most studied parasites due to its medical and veterinary importance, its wide range of distribution and its suitability as a model of study in pharmacogenomics, cell biology, molecular genetics and immunology. *T. gondii* infections are generally subclinical in healthy individuals but can be major problems for immunosuppressed adults and fetuses (Dubey, 2008). The severity of such infections can vary greatly, perhaps based on the status of the host immune system (Lahmar et al., 2009), the genotype of the infective parasite strain (Ferreira et al., 2011) and the host's genetic background (Sullivan and Jeffers, 2012). There has been significant progress, but no vaccine is currently available that will prevent *T. gondii* infection; indeed, very few drugs effectively reduce *T. gondii*'s presence in infected individuals (Zhou et al., 2016a). Researchers frequently select models for studying *T. gondii* based on their similarity to humans in terms of genetics, anatomy, and physiology; this includes the cellular models that have been used to study *T. gondii* (Szabo and Finney, 2017). However, many studies make use of mouse models (Alfonzo et al., 2002; Martens et al., 2005; Tanaka et al., 2013; Unno et al., 2013; Dzitko et al., 2015; He et al., 2015). There have been highly controversial results associated with some of these models because they do not completely mimic human toxoplasmosis (Hunter and Sibley, 2012; Niedelman et al., 2012; Seok et al., 2013). In fact, some scientists have argued that new approaches must be explored; some have even proposed new models for studying *T. gondii* (Cornelissen et al., 2014; Tanaka et al., 2016; Nau et al., 2017).

**Figure 1:**
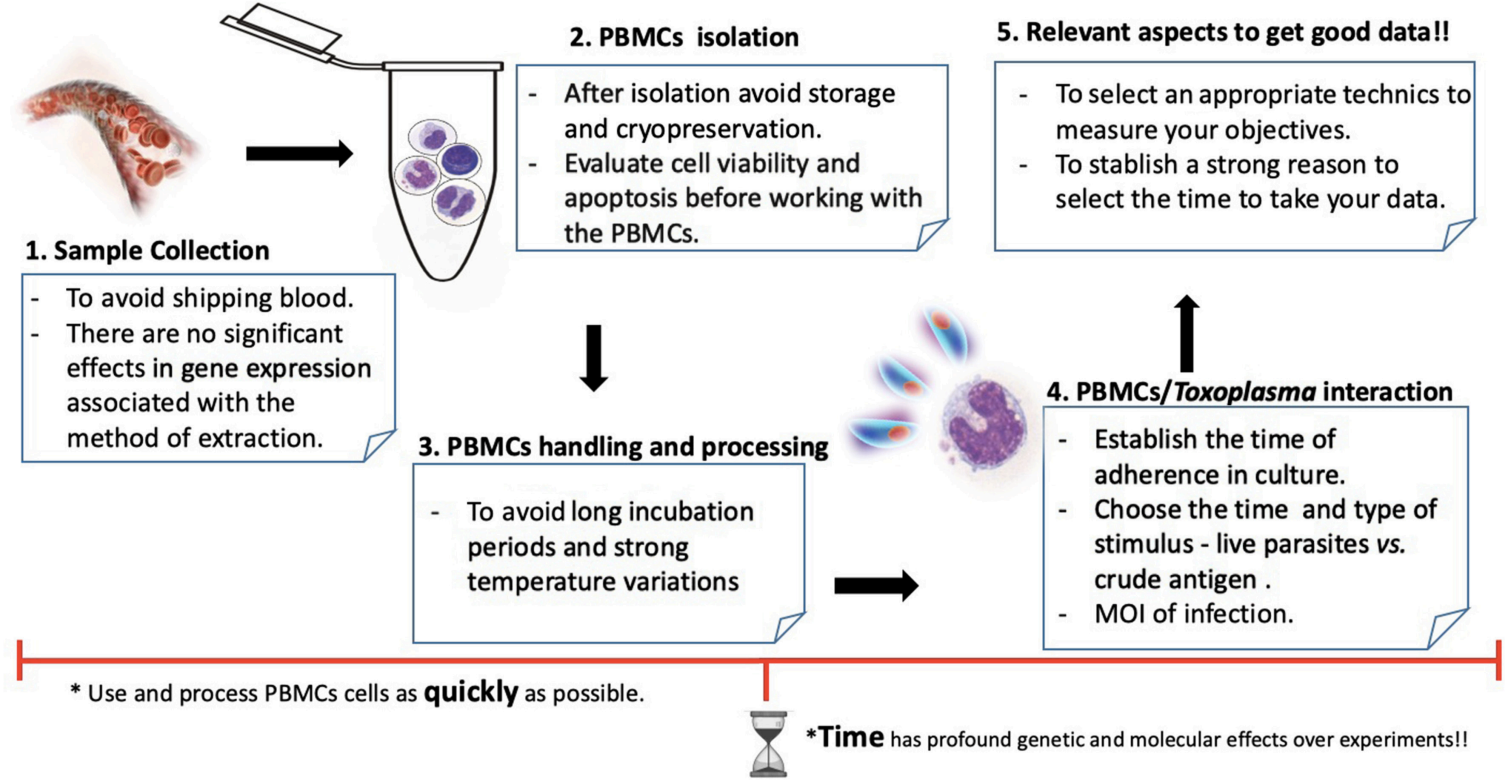
Workflow and aspects to keep in mind to work with PBMCs and *Toxoplasma gondii*.

The PBMC cellular model includes T and B cells (~80%), natural killer cells (~10%) and monocytes (~10%) (Autissier et al., 2010). These blood cells play an important role in the immune response that is meant to preserve the host's homeostasis and defend it against parasite infection (Zhou et al., 2016a). Researchers have used PBMC to study *T. gondii* with various goals, but especially to improve diagnostic, drug-screening and immunogenetic approaches (Vendrell et al., 1992; Dzitko et al., 2015). Although PBMCs do not completely mimic an infection *in vivo*, they can be taken directly from affected individuals and can generate certain qualities that when added to the recommendations (Figure 1) discussed below can improve the quality of the experimental data regarding toxoplasmosis. Therefore, understanding and improving models is imperative to the appropriate interpretation and translation of this work into clinical setting (Szabo and Finney, 2017). In this review, we present a summary of how PBMCs have been used to study *T. gondii* and other apicomplexan parasites, discuss some controversies related to this cellular model and then describe possible improvements to the related protocols.

## Immune Response in PBMCs Stimulated With *T. gondii*

PBMCs have mainly been used to model *T. gondii* as part of the evaluation of potential new vaccines or drugs, as well as to understand the relationships between the host's immune system and the parasite (see Table 1). The studies using these models have shown that cytokine levels can vary according to the evaluated clinical condition, the type of strain and the culture conditions which can include the type of media culture supplement; the time of data collection; and the temperature variations during storage, shipping and handling (Weinberg et al., 2010). In chronic asymptomatic individuals, the PBMCs' immune responses against total lysate antigen and against peptides derived from *T. gondii* are predominantly characterized by high levels of interferon gamma (IFN-γ) (Prigione et al., 2006; Bayram Delibaş et al., 2009; Cong et al., 2011; Cardona et al., 2015; Meira et al., 2015); in ocular toxoplasmosis and *Toxoplasma*-seronegative individuals, however, the level of this cytokine is much lower (Alfonzo et al., 2005; Meira et al., 2014; Maia et al., 2017). PBMCs have also been useful in studying the immune response of HIV-infected individuals and of pregnant women with toxoplasmosis. In one study on HIV patients who had been coinfected with *T. gondii*, researchers measured the IFN-γ expression of stimulated total lysate antigen using PBMCs, both before and after treatment with antiparasitic drugs (sulfadiazine, pyrimethamine, folinic acid, trimethoprim-sulfa-methoxazole, and corticosteroids); the infection's evolution was correlated with the restoration of the IFN-γ response and with decreased inflammation (Meira et al., 2015). In another study, researchers showed that, during pregnancy, tumor necrosis factor alpha (TNF-α) and interleukin (IL)-12 had decreased expression when cells were stimulated with live tachyzoites (Rezende-Oliveira et al., 2012). Interestingly, the addition of the prolactin hormone to the cells seemed to restrict the parasite's proliferation (Dzitko et al., 2012). In similar works, researchers have shown the importance of IFN-γ production in the congenital transmission of *T. gondii* through the upregulation of intercellular adhesion molecular 1 (ICAM-1) (Pfaff et al., 2005). Stimulating PBMCs with complete or partial antigens of *T. gondii* seems to reveal important aspects of the host's immune response. However, *T. gondii* and other apicomplexans secrete proteins in a highly regulated manner that is involved in the parasite's immune evasion mechanisms (Tosh et al., 2016). These processes are not seen when parasite antigens are used, so we recommend the use of live parasites to stimulate PBMCs (Figure 1).

**Table 1:** Characteristics and use of PBMC as a study model for *T. gondii* and other Apicomplexan parasites during the last 10 years.

**Organism**	**Technical observations**	**PBMC culture (number of cells/final volume/culture plates)**	**Main findings**	**References**
Human PBMC and *Toxoplasma gondii*	**Stimulus:** RH strain (TLA, 1 μg/mL) **Technic:** RT-qPCR **Process time:** 48 h after collection **Cryopreserved:** No **Supplemented:** 10% FCS	1 × 10^6^/500 μL/48-well	High levels of TGF-β, IL-6, IL-10 in OT individuals	Maia et al., 2017
	**Stimulus:** RH strain (TLA, 1 μg/mL) **Technic:** RT-qPCR **Process time:** 48 h after collection **Cryopreserved:** No **Supplemented:** 10% FBS	1 × 10^6^/500 μL/48-well	TATA box-binding protein (TBP) and ubiquitin C (UBC) are the most stable genes for mRNA analysis in PBMCs	Meira-Strejevitch et al., 2017
	**Stimulus:** ND **Technic:** Radioactivity **Process time:** 168 h in incubation **Cryopreserved:** No **Supplemented:** 5% human AB serum	ND	Despite high proliferation, lymphocytes from meth users had a lower proliferative capacity	Massanella et al., 2015
	**Stimulus:** Peptides from P30 and ROP 18 (10 μg/mL) **Technic:** ELISPOT **Process time:** 24 h in incubation **Cryopreserved:** Yes **Supplemented:** No	2 × 10^5^/100 μL/96-well	Four peptides induced IFN-γ expression	Cardona et al., 2015
	**Stimulus:** RH strain (TLA, 1 μg/mL) **Technic:** ELISA **Process time:** 48 h in incubation **Cryopreserved:** No **Supplemented:** 10% FBS	1 × 10^6^/500 μL/48-well	Restoration of IFN-γ response and a decrease of the inflammatory cytokines TNF-α and IL-10	Meira et al., 2015
	**Stimulus:** RH strain (Live) **Technic:** Radioactivity **Process time:** ND **Cryopreserved:** ND **Supplemented:** ND	2.5 × 10^5^/100 μL/96-well	Phytoecdysteroids did not inhibit *Toxoplasma* and did not affect the cytokine response (IFN-γ. IL-12, IL-10)	Dzitko et al., 2015
	**Stimulus:** TLA (ND) **Technic:** ELISPOT **Process time:** 120 h in incubation **Cryopreserved:** No **Supplemented:** No	3 × 10^5^/200 μL/ND	No association was observed when PBMCs were stimulated with TLA or mitogen	Nogueira et al., 2014
	**Stimulus:** BK strain (Live) **Technic:** ELISA **Process time:** 48 h in incubation **Cryopreserved:** No **Supplemented:** 0.1% BSA	2.5 × 10^6^/ND/ND	Correlation between Prolactine and the level of IL-10, but not with IFN-γ	Dzitko et al., 2012
	**Stimulus:** RH and ME49 strains (Live) **Technic:** ELISA **Process time:** 48 h in incubation **Cryopreserved:** No **Supplemented:** No	2 × 10^6^/ND/24-well	*T. gondii*-seronegative non-pregnant women produced significantly higher levels of TNF-a and IL-12	Rezende-Oliveira et al., 2012
	**Stimulus:** *T. gondii* peptides **Technic:** ELISPOT **Process time:** ND **Cryopreserved:** Yes **Supplemented:** No	2 × 10^5^/100 μL/96-well	Peptides induced significant IFN-γ production by PBMCs from 4 HLA-A*0201 persons infected with T. gondii	Cong et al., 2011
	**Stimulus:** TRRH strain (TLA, 5 mg/mL) **Technic:** ELISA **Process time:** 72 h in incubation **Cryopreserved:** No **Supplemented:** 10% FBS	1 × 10^6^/200 μL/96-well	IL-5 was higher than IFN-γ in the initial phase of the infection; as the IgG started to rise, IFN-γ increased and suppressed the synthesis of IL-5	Bayram Delibaş et al., 2009
Pigs PBMC and *Toxoplasma gondii*	**Stimulus:** IPB-G/LR strain (TLA, 10 μg/mL) **Technic:** Flow cytometry **Process time:** 72 h in incubation **Cryopreserved:** No **Supplemented:** 10% FBS	1 × 10^6^/ND/ND	High levels of IFN-γ	Jennes et al., 2017
	**Stimulus:** RH strain (TLA) **Technic:** RNAseq **Process time:** 8, 24, 48 h in incubation **Cryopreserved:** No **Supplemented:** 10% FBS	ND	More than 2,400 differentially expressed genes	Zhou et al., 2016a
Human PBMC and *Plasmodium* sp.	**Stimulus:** *P. falciparum* crude lysate (50 mg) **Technic:** Flow cytometry and RT-qPCR **Process time:** 168 h in incubation **Cryopreserved:** No **Supplemented:** 10% FCS	1 × 10^6^/ND/ND	Crude antigens exhibited strong heterogeneity in the cytokine production	Kijogi et al., 2018
	**Stimulus:** *P. falciparum* crude lysate (50 mg) **Technic:** Flow cytometry and RT-qPCR **Process time:** 10 h in incubation **Cryopreserved:** No **Supplemented:** 2% FCS	ND	Decreased parasite growth and expression of PD-1 and IL-10 genes using L-citrulline supplemented media	Awasthi et al., 2017
	**Stimulus:** Peptide Pooling Scheme **Technic:** ELISPOT **Process time:** 18 h in incubation **Cryopreserved:** ND **Supplemented:** 10% FCS	1 × 10^6^/ND/96-well	Highest immunogenicity was identified at 7 days after boosting with 932 SFC compared with 57 SFC among control vaccinees	Mensah et al., 2016
	**Stimulus:** Recombinant RAMA protein **Technic:** ELISA **Process time:** ND **Cryopreserved:** ND **Supplemented:** ND	ND	High levels of interferon (IFN)-γ and interleukin (IL)-10 cytokines were detected	Changrob et al., 2016
	**Stimulus:** Peptides from CSP and AMA1 protein (10 μg/ml) **Technic:** ELISPOT **Process time:** 36 h in incubation **Cryopreserved:** No **Supplemented:** No	1 × 10^6^/100 μL/96-well	CSP and AMA1 peptides recalled IFN-γ responses from naturally exposed individuals	Ganeshan et al., 2016
	**Stimulus:** TLR1/2 ligand PAM3CSK4 (20 ng) **Technic:** ND **Process time:** 72 h in incubation **Cryopreserved:** Yes **Supplemented:** ND	2 × 10^5^/100 μL/96-well	IL-1β and TNF-α were significantly higher in severe malaria cases compared with healthy controls	Manning et al., 2016
	**Stimulus:** CelTOS (10 ng) or other single (1.25 μg/ml) peptide pools **Technic:** ELISPOT **Process time:** 36 h in incubation **Cryopreserved:** No **Supplemented:** No	4 × 10^5^/100 μL/96-well	Natural malaria transmission induces CelTOS-specific *ex vivo* IFN-γ	Anum et al., 2015
Calves PBMC and *Cryptosporidium parvum*	**Stimulus:** Recombinant *C. parvum* p23 vaccine antigen **Technic:** Flow Cytometry **Process time:** ND **Cryopreserved:** ND **Supplemented:** ND	ND	Recombinant p23 vaccine antigen can stimulate a Type-1-like immune response	Wyatt et al., 2005
Human PBMC and *Eimeria* sp.	**Stimulus:** Recombinant Eimeria Antigen (rEA) **Technic:** Flow Cytometry **Process time:** 72 h in incubation **Cryopreserved:** ND **Supplemented:** 5%FCS	25 × 10^6^/ND/6-well	rEA stimulates human NK cell effector functions including increasing levels of IFN-γ and Granzyme B	Aylsworth et al., 2013
Sheep PBMC and *Babesia* sp.	**Stimulus:** BdE or BQ1E (10 μg/well) **Technic:** ELISA **Process time:** 120 h in incubation **Cryopreserved:** No **Supplemented:** 10% autologous plasma	2 × 10^5^/ND/96-well	Production of IFN-γ and IL10 have key roles in the course of infection by *Babesia* sp.	Guan et al., 2010
Bovines PBMC and *Theileria* sp.	**Stimulus:** Sporozoites in homogenized infected tick **Technic:** RT-qPCR **Process time:** 48 h in incubation **Cryopreserved:** No **Supplemented:** 40% FBS	4 × 10^6^/ND/6-well	MHC-DQ, SIRPA, PRNP, TLR10, cMAF and MAFB genes showed no change in mRNA expression after *T. annulata* infection	Panigrahi et al., 2016
	**Stimulus:** Sporozoites in homogenized infected tick **Technic:** RT-qPCR **Process time:** 48 h in incubation **Cryopreserved:** No **Supplemented:** 40% FBS	2 × 10^6^/ND/6-well	Up-regulation in SIRPA, PRNP and MHC DQα genes and down-regulation in TLR10, cMAF and MAFB genes in crossbreds as compared to indigenous cattle was observed	Dewangan et al., 2015
	**Stimulus:** MPSP Peptides **Technic:** ELISPOT **Process time:** 42 h in incubation **Cryopreserved:** Yes **Supplemented:** No	1 × 10^6^/100 μL/96-well	IFN-γ and IL-10 were detected in infected Holsteins but weak responses were exhibited by infected Angus and Japanese Black cattle	Yamaguchi et al., 2010

*TLA, T. gondii Lysate Antigen; ND, Not determine; OT, Ocular Toxoplasmosis; NC, Negative Control; FCS, Fetal Calf Serum; MPSP, Major piroplasm surface protein; RAMA, Rhoptry-associated membrane antigen; SFC, Spot Forming Cells (SFC)/106; CSP, Plasmodium falciparum circum- sporozoite protein; AMA-1, Apical membrane antigen-1*.

On the other hand, the vaccine candidates for *T. gondii* are typically parasite proteins or the peptides that elicit protective immune responses in mice. However, vaccine candidates that are effective in mice are not necessarily effective in humans. PBMCs are potentially very useful tools for identifying and characterizing novel vaccine candidates for *T. gondii*. Cells from individuals with varied genetic and immunological backgrounds can be easily isolated and stimulated with the antigens of interest, thus allowing measurement of the desired cytokine profile or cell response. However, to our knowledge, few researchers have used this strategy (Tan et al., 2010; Cong et al., 2012; Cardona et al., 2015). The studies mentioned above have identified novel parasite derived peptides that induce strong production of IFN-γ in people who express one of the most common human leukocyte antigen (HLA) supertypes (HLA-A02, HLA-B07, and HLA-A11), making those peptides attractive vaccine candidates. PBMCs have also been used to evaluate the efficacy of two phytoecdysteroids (α-ecdysone and 20-hydroxyecdysone) in controlling *T. gondii* infections. These drugs are effective against *Babesia gibsoni* but have no effect on *T. gondii*'s proliferation and do not elicit a Th1 protective immune response against the parasite (Dzitko et al., 2015). Thus, all these studies show that PBMCs may represent an important and undervalued model for vaccine and drug development.

## Technical Aspects to Consider Before Working With PBMCs and *T. gondii*

To obtain scientifically valid data, the experimental conditions and the study's model must be closely controlled. PBMCs are one of the best sources for assessing the differences or changes associated with diseases or with drug responses and therapies; in addition, these cells are relatively easy to obtain from whole blood through isolation (Burczynski and Dorner, 2006). A major challenge in the monitoring of PBMCs' quality is establishing protocols that define the proper isolation, shipping and storage methods so that they can be tested without changes in cellular functionality. For researchers who work with PBMCs, it is very important to note that many variables affect these cells. This model can be used to study Toxoplasma under clinical or non-clinical conditions; however, the whole procedure—from blood withdrawal to experimentation—must be highly standardized. PBMCs are perishable living cells, and some of them begin to die immediately after their isolation from whole blood. Scientists have compared isolation techniques, but they have found no differences with respect to the generally used methods, which include Ficoll-Paque density-gradient centrifugation and BD Vacutainer cell-preparation tubes (Corkum et al., 2015).

However, after isolation of PBMC, early apoptotic events are present in both *in vitro* and *ex vivo* experiments; as a result, it is very important to determine apoptosis before using these cells in the experiments (Wunsch et al., 2015). Various methods have been used to measure apoptosis, including the YO-PRO-1/7-AAD method, which has been proposed as a good, low-cost alternative for sensitive detection of early apoptosis in PBMCs and >80% of viability is recommended before starting to work (Glisic-Milosavljevic et al., 2005). Working with freshly isolated PBMCs is not always possible; thus, the cells are generally frozen and thawed for processing at later times (see Table 1); this allows for the batched thawing of samples and for direct comparability in assays, thus reducing inter-assay variability and allowing for future analysis of later-emerging issues (Riedhammer et al., 2016). As a consequence, both apoptosis and necrosis happen; this phenomenon has been well documented to occur during cryopreservation (Fowke et al., 2000; Baust, 2002; Cosentino et al., 2007; Mallone et al., 2011). For example, in a recent study on how storage temperature affects PBMCs and cryopreservs PBMCs' viability, recovery and gene expression patterns were all affected, as compared to those of freshly isolated PBMCs (Yang et al., 2016). In a cell infected with *Toxoplasma*, each hour represents a specific differential gene-expression profile (He et al., 2015; Zhou et al., 2016b), which indicates that the best possible method for handling missing data is to prevent the problem by properly planning each study and by collecting the data carefully (Wisniewski et al., 2006). The goal is to eventually have studies with comparable data. Along the same order of ideas, the time variable is important to consider when working with PBMCs, as it can lead to missing data. The problem of missing data is relatively common in most fields of research, and it can significantly affect the conclusions drawn from the data (Little et al., 2012). Accordingly, some medical researchers have focused on handling missing data and related problems using methods that prevent or minimize missing data (O'Neill and Temple, 2012). One of the main problems with using the PBMC model for T. gondii is that the majority of studies are not comparable, as researchers do not usually consider the time aspect when obtaining data. Most studies have shown that the time spent collecting data from cells after a stimulus varies; for example, no one has argued that a supernatant for cytokine measurement should be performed in an exact period of time. In the bulk of the studies, there are differences in the time accorded to the PBMC culture (varying from 48 h to 7 days) and even in the collection of supernatant for the measurement of IFN-γ after 12, 24 or 48 h (see Table 1). In this sense, the time between the making of the culture and the collection of data should be methodologically explained.

## PBMCs in Other Apicomplexan Parasites

Although apicomplexans comprise a large phylum of parasitic organisms (with more than 5,000 species), only a few have been studied in detail. Most of these studies focus on parasites that produce disease in humans such as *T. gondii, Plasmodium* spp. and *Cryptosporidium*. There are fewer studies on parasites that do not affect humans directly (e.g., *Eimeria, Babesia*, and *Theileria*). One of the most studied apicomplexan parasites is *Plasmodium*, the pathogen that causes malaria, one of the most important public health problems worldwide. Nearly all studies regarding this parasite that have used PBMCs have focused on the development of vaccine candidates, particularly those using the peptide polling scheme (Anum et al., 2015; Ganeshan et al., 2016; Mensah et al., 2016) or recombinant proteins (Garraud et al., 2002; Garg et al., 2008; Gitau et al., 2014; Changrob et al., 2016). These studies have shown that, when PBMCs are challenged with molecules derived from *Plasmodium*, the immune response is characterized by Th1 cytokines such as IFN-γ, IL-1β, and TNF-α. The nuclear transcription factor kappa B (NF-kB) is what mainly regulates these proinflammatory cytokines. However, patients with complications of malaria have much lower levels of NF-kB than healthy controls do; as a consequence, these low levels limit the Th1 cell response (Punsawad et al., 2012).

Similarly, low levels of IL-1β and TNF-α have been found in patients with severe malaria. In one study, researchers analyzed 29 single-nucleotide polymorphism (SNPs) in the PBMCs of patients with various clinical conditions and found that only the “toll-like receptor-1” variant could contribute to this reduced cellular phenotype (Manning et al., 2016). Although this and other studies have shown that malaria infections inhibit the immune response, in one recent study, PBMCs from asymptomatic school children showed a strong heterogeneity of cytokine production, which suggests suppress immune responses could be related only with active infections (Kijogi et al., 2018). The donors' immunological histories could influence this strong heterogeneous response because these PBMCs could present cross-reactivity with other infections such as schistosomiasis, leishmaniosis, toxoplasmosis, and Chagas disease. For this reason, other authors have proposed alternatives to the use of PBMCs in studying the primary immune response; one such alternative are hematopoietic stem cells (naïve cells), which would reduce the discrepancies in mononuclear cell quality between studies (Chitsanoor et al., 2017).

In a study of *Cryptosporidium parvum* in calves recovering from cryptosporidiosis, scientists used PBMCs to evaluate the immunogenic potential of a specific protein (p23) as a vaccine antigen; in the first step, the researchers infected the calves with the parasite's oocysts, and in the next step, the PBMCs from the calves were stimulated with recombinant p23, showing that this antigen can stimulate a Type-1-like immune response among T cells (Wyatt et al., 2005). A similar response occurred in HIV-positive people infected with *Cryptosporidium*; their PBMCs were used to evaluate the cytokine profile after stimulation with the parasite crude extract, and this showed that INF-γ is one of the most important cytokines in the immune response against this parasite (Gomez Morales et al., 1999). With the help of IL-15, INF-γ eliminates this intracellular parasite by activating natural killer cells (Dann et al., 2005). In the case of *Eimeria*, PBMCs have been used to evaluate the parasite's proteins as adjuvants for vaccines or for immunostimulatory therapeutic agents in the treatment of human cancer (Aylsworth et al., 2013). For *Babesia*, PBMCs have been utilized to evaluate the cytokine profile response in cattle vaccination (East et al., 1997) and to identify the immune mechanisms involved in this parasite's pathogenicity (Guan et al., 2010).

With respect to *Theileria*, researchers have evaluated the mRNA levels of six immunological markers in PBMCs from crossbred, Tharparkar and Buffalo cattle after a parasite challenge. The markers included MHC class II DQ-α (BoLA-DQ), signal-regulatory protein alpha, prion protein, toll-like receptor 10, c-musculoaponeurotic fibrosarcoma oncogene homolog and V-maf avian musculoaponeurotic fibrosarcoma oncogene homolog B. For crossbred and Tharparkar cattle, significant differences occurred in the expression of genes in infected and uninfected cells (Dewangan et al., 2015), whereas the genes in Buffalo cattle did not show significant differences, suggesting that those genes had little effect in the progression of tropical theileriosis in the Buffalo species (Panigrahi et al., 2016). These results indicate that, although PBMCs are a good model for studying immunological phenomena, using them to make inferences or generalize results across species is not advisable, regardless of how evolutionarily close those species are.

The evidence suggests that PBMCs could be a good model for studying the immune response in apicomplexan parasites and for evaluating the efficiency of vaccine candidates, therapeutic agents and immunomodulatory molecules. However, these cells are not the most appropriate model for studying primo-infections (Chitsanoor et al., 2017). Therefore, despite all the benefits of the PBMC model, precautions should be taken related to its limitations and the type of immune response that is being evaluated.

## PBMCs, Omics, and *Toxoplasma*

Omics techniques are powerful tools in modern biology, as they enable high-throughput measurements of many genes, proteins and metabolites in samples. A limited number of studies with Toxoplasma have employed PBMCs in global transcriptional profiling, with only one using PBMCs from pigs (Zhou et al., 2016a; see Table 1). Some other studies have applied omics in research on Toxoplasma and cortical neurons, astrocytes, skeletal muscle cells, fibroblasts, and other cells; almost all of these were derived from murine models (Tanaka et al., 2013; Pittman et al., 2014; He et al., 2016; Zhou et al., 2016b). All these studies show that gene expression—and consequently, protein and metabolite levels—can undergo changes based on physiological conditions. The results of other studies suggest that the gene expression patterns in PBMCs greatly depend on temporal and interindividual variations and also show strong evidence that these cells' gene-expression profiles are very sensitive to long incubation periods (Baechler et al., 2004) in addition to being dramatically affected by cryopreservation (Yang et al., 2016). As yet, no reports in the literature exist regarding the analysis of global transcriptional profiling using human PBMCs stimulated with *T. gondii*. Therefore, omics techniques, including dual RNAseq (Westermann et al., 2017) in PBMCs from healthy individuals and toxoplasmosis sufferers, could provide excellent opportunities to obtain important and relevant data on this infectious disease. Given the number of methodological differences among studies, *ex vivo* assays can be considered a suitable model for the analysis of what occurs during infection with Toxoplasma in humans. These assays emerge as a good alternative for these main reasons: (i) an *ex vivo* experiment should usually be done within a 24 h period in order to minimize the effect that stress generates on the cells; (ii) PBMCs' characteristics are very interesting because they are primary cells, are not immortalized and have the genetic backgrounds of real patients; and (iii) *ex vivo* experiments have the advantage of being analyzable very shortly after sampling, which is particularly critical in studies of gene expression, where data can be altered dramatically with the time factor.

## Conclusion

PBMCs allow for the study of the immune-system response to infections with apicomplexan parasites, the evaluation of vaccine candidates and the development of immunotherapeutic strategies. To obtain reproducible and comparable results, several variables must be optimized when working with this model. The evaluated response of human PBMCs in apicomplexans has also been restricted to a few cytokines, so it is highly advisable to include additional immunological techniques that can comprehensively reflect the host's response to the pathogen.

## Author Contributions

JA and AH contributed to the conception of the publication and prepared the manuscript. Both authors read and approved the final manuscript.

### Conflict of Interest Statement

The authors declare that the research was conducted in the absence of any commercial or financial relationships that could be construed as a potential conflict of interest.
